# Inhibitors of ribosome biogenesis repress the growth of *MYCN*-amplified neuroblastoma

**DOI:** 10.1038/s41388-018-0611-7

**Published:** 2018-12-12

**Authors:** Øyvind H. Hald, Lotte Olsen, Gabriel Gallo-Oller, Lotta Helena Maria Elfman, Cecilie Løkke, Per Kogner, Baldur Sveinbjörnsson, Trond Flægstad, John Inge Johnsen, Christer Einvik

**Affiliations:** 1 Department of Pediatrics, Division of Child and Adolescent Health, UNN – University Hospital of North-Norway, NO-9038 Tromsø, Norway; 2 Pediatric Research Group, Department of Clinical Medicine, Faculty of Health Science, The Arctic University of Norway – UiT, NO-9037 Tromsø, Norway; 3 Childhood Cancer Research Unit, Department of Women’s and Children’s Health, Karolinska Institutet, 171 76 Stockholm, Sweden; 4 Molecular Inflammation Research Group, Department of Medical Biology, Faculty of Health Science, The Arctic University of Norway – UiT, NO-9037 Tromsø, Norway

**Keywords:** Paediatric cancer, Targeted therapies

## Abstract

Abnormal increases in nucleolar size and number caused by dysregulation of ribosome biogenesis has emerged as a hallmark in the majority of spontaneous cancers. The observed ribosome hyperactivity can be directly induced by the *MYC* transcription factors controlling the expression of RNA and protein components of the ribosome. Neuroblastoma, a highly malignant childhood tumor of the sympathetic nervous system, is frequently characterized by *MYCN* gene amplification and high expression of *MYCN* and *c-MYC* signature genes. Here, we show a strong correlation between high-risk disease, *MYCN* expression, poor survival, and ribosome biogenesis in neuroblastoma patients. Treatment of neuroblastoma cells with quarfloxin or CX-5461, two small molecule inhibitors of RNA polymerase I, suppressed MycN expression, induced DNA damage, and activated p53 followed by cell cycle arrest or apoptosis. CX-5461 repressed the growth of established *MYCN*-amplified neuroblastoma xenograft tumors in nude mice. These findings suggest that inhibition of ribosome biogenesis represent new therapeutic opportunities for children with high-risk neuroblastomas expressing high levels of Myc.

## Introduction

Neuroblastoma, a childhood tumor of the peripheral sympathetic nervous system, originates from neural crest cells and usually manifests in the adrenal gland or in a paraspinal location in the abdomen or chest. The clinical features of neuroblastoma are characterized by heterogeneity spanning from spontaneous regression or differentiation with an overall survival of 85–90%, to treatment-refractory progression and metastatic tumors with less than 50% of the patients surviving despite intensive therapies [1]. Currently, no effective therapy exists for patients with recurrent or relapsed neuroblastoma, and new treatment modalities for these patients are urgently needed.

A molecular hallmark of high-risk neuroblastoma is genetic amplification and high expression of the *MYCN* oncogene [2]. Also, single-copy high-risk neuroblastomas frequently show high expression of the *MYCN* homolog *c-MYC* [3]. The MycN and c-Myc proteins are transcription factors, and exert their oncogenic effects through the activation and repression of a wide array of genes controlling fundamental cellular processes, including proliferation, cell growth, metabolism, differentiation, and migration [4].

Ribosomal biogenesis is upregulated in malignant cells, and nucleolar enlargement has been used as a marker for the histopathological diagnosing of cancer for over a century [5]. MycN has been shown to positively regulate the expression of a large set of genes involved in ribosomal biogenesis [6], and also c-Myc is well-established as a driver of this process [7]. In line with these observations, tumor cells from *MYC*-driven neuroblastomas frequently display nucleolar hypertrophy [8, 9]. In recent years, specific inhibitors of ribosomal biogenesis have been developed and characterized. Two small molecular compounds, quarfloxin (CX-3543) and CX-5461, target and inhibit RNA pol I activity. CX-5461 is currently being tested in patients with advanced solid tumors (NCT02719977) and advanced hematological cancer (ACTRN12613001061729) [10]. Quarfloxin has completed phase 1 and 2 trials in patients with advanced solid tumors and lymphomas (NCT00955786) and neuroendocrine/carcinoid tumors (NCT00780663), respectively. Inhibition of RNA pol I activity has been shown to induce apoptosis, nucleolar surveillance signaling, p53 pathway activation, senescence, and pro-death autophagy [11–14].

In this study, we demonstrate a strong correlation between advanced stage disease, high *MYCN* expression levels, and elevated expression of genes involved in ribosome biogenesis in several large neuroblastoma patient cohorts. Based on these observations, we evaluated the effects of quarfloxin and CX-5461, two small molecule inhibitors of ribosome biogenesis in neuroblastoma cell lines and xenografts. Both quarfloxin and CX-5461 are cytotoxic to neuroblastoma cells in nanomolar concentrations and orally administered CX-5461 represses the growth of *MYCN*-amplified neuroblastoma xenografts in mice. Mechanistically, we demonstrate that both compounds induce p53 signaling, cell cycle arrest, DNA damage, and apoptosis of neuroblastoma cells and reduced MycN and RNA pol I activity.

## Results

### Neuroblastoma tumors with high ribosome biogenesis activity have poor a prognosis

**Fig. 1:**
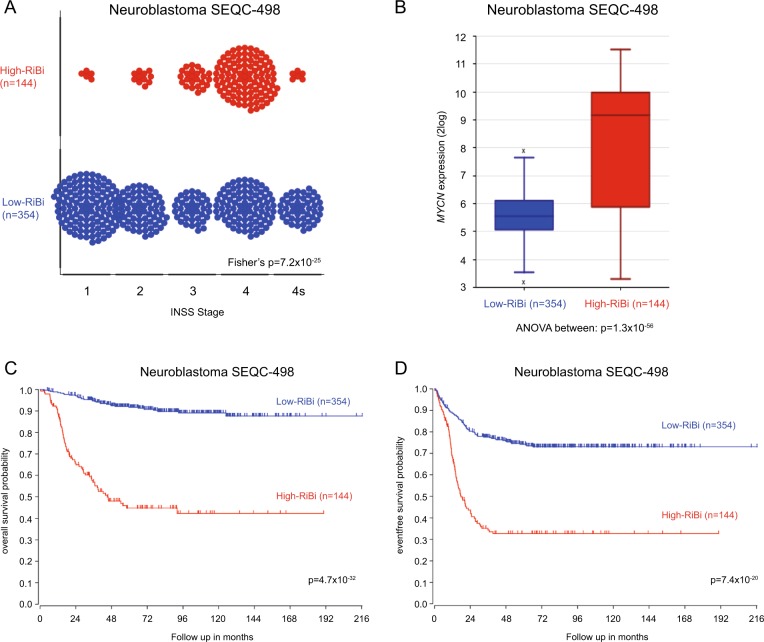
Neuroblastoma tumors with enhanced ribosome biogenesis activity are characterized by high *MYCN* expression, advanced stage disease, and poor prognosis. **a** Plot showing the distribution of High-RiBi and Low-RiBi neuroblastoma tumors in different INSS stages. **b** Boxplot showing *MYCN* expression in tumors defined by High-RiBi and Low-RiBi. High-RiBi tumors show significantly higher *MYCN* expression. Kaplan–Meier analysis showing overall **c** and event-free **d** survival of neuroblastoma patients defined by High-RiBi and Low-RiBi tumors. The analyses were performed on publically available data (Tumor Neuroblastoma SEQC-498-RNAseq) from R2: Genomic Analysis and Visualization Platform (http://r2.amc.nl)

A previous study by Boon et al. showed that the MycN protein enhances the rate of ribosome biogenesis in neuroblastoma cell lines [6]. To investigate how genes regulating ribosome biogenesis correlate with clinical parameters in neuroblastoma, we performed an unsupervised clustering analysis (k-means clustering) to subdivide the tumors in a large neuroblastoma RNAseq dataset (SEQC-498) in two groups according to expression of genes defined by the KEGG pathway “Ribosome Biogenesis in Eukaryotes”. The 498 neuroblastoma tumors clustered into two well-defined groups characterized by low (Low-RiBi, *n* = 354) and high (High-RiBi, *n* = 144) expression of ribosome biogenesis genes (Supplementary Figure 1). Eighty-five percent of tumors defined by the High-RiBi group belong to advanced stage disease (INSS 3 and 4) (Fig. 1a). Furthermore, the High-RiBi tumors were characterized by high *MYCN* expression (Fig. 1b). Kaplan–Meier analyses of the two clusters showed that tumors from the High-RiBi group had a very poor overall- and event-free survival (log-rank test, *p* = 4.7 × 10^−32^ and *p* = 7.4 × 10^−20^, respectively, Figure 1c, d). Similar results were observed for several independent neuroblastoma cohorts investigated (Supplementary Figure 2A–D). These data demonstrate that neuroblastoma tumors with enhanced ribosome biogenesis activity are characterized by high *MYCN* expression, advanced stage disease, and poor prognosis.

### Inhibitors of ribosome biogenesis decrease neuroblastoma cell viability

**Fig. 2:**
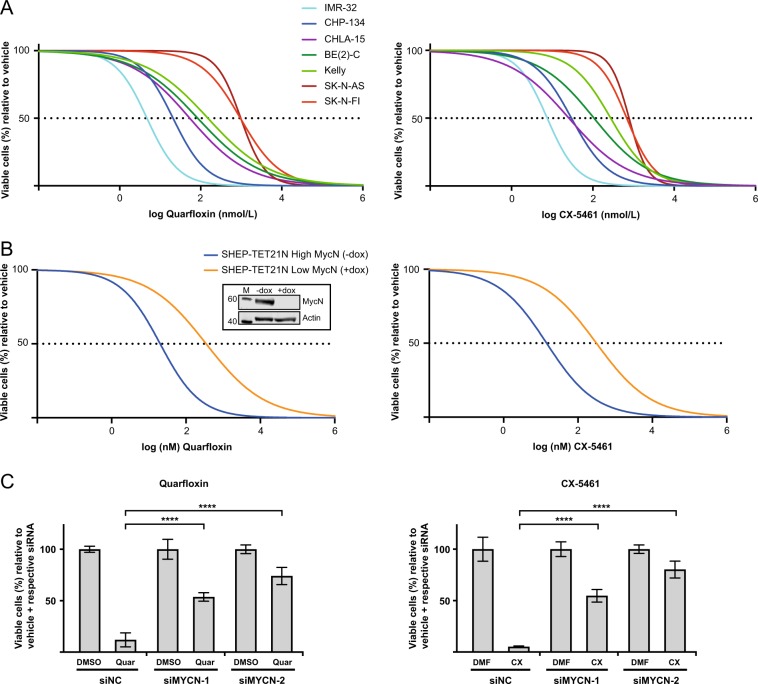
Cell viability of neuroblastoma cell lines treated with quarfloxin or CX-5461. **a** Cell viability of neuroblastoma cell lines treated with an 8-log fold dose range of quarfloxin (left panel) or CX-5461 (right panel). Absolute half-maximal inhibitory concentrations (IC_50_ values) are shown in Table 1. **b** SHEP-TET21N cells were seeded in the presence (low MycN) or absence (high MycN) of 1 ug/mL doxycycline (dox). On the following day, cells were treated for 48 h with an 8-log fold change dose range of quarfloxin (left panel) or CX-5461 (right panel). IC_50_ values are shown in Table 1. Insert: WB showing MycN expression in absence (-dox) and in presence of dox ( + dox). M = marker. Numbers to left indicate MW in kDa. **c** Cell viability of IMR-32 cells transfected with siRNAs (siMYCN-1 and siMYCN-2) targeting *MYCN* or a negative control siRNA (siNC), and treated with 50 nM quarfloxin (left panel) or 75 nM CX-5461 (right panel) for 48 h. The viability of vehicle + respective siRNA was set to 100%, and quarfloxin and CX-5461 treated cells were normalized to their respective controls. DMSO and DMF are vehicle controls to quarfloxin and CX-5461, respectively. For a, b, c; cell viability was measured with the Alamar blue assay. The data represents the mean cell viability and SD of two individual experiments performed in duplicate. (***p ≤ 0.001; ****p ≤ 0.0001)

Given that the expression of genes involved in ribosome biogenesis strongly correlated with neuroblastoma high-risk disease and prognosis, we evaluated the effects of two compounds inhibiting RNA polymerase I in a panel of neuroblastoma cells (Supplementary Table 1). Neuroblastoma cells were incubated with an 8-log dose range of CX-5461 (0.0005–5000 nM) or quarfloxin (0.001–10000 nM) for 48 h (Fig. 2a), and absolute IC_50_ values were calculated (Table 1). *MYCN*-amplified (MNA) and wild-type *TP53* (wt-*TP53*) IMR-32 and CHP-134 cells, and *c-MYC* overexpressing/wt-*TP53* CHLA-15 cells, were highly sensitive to the action of both drugs. Also, the IC_50_ of MNA/mut-*TP53* cell lines BE(2)-C and Kelly were substantially lower than those of non-MNA/mut-*TP53* SK-N-AS and SK-N-FI cells.

**Table 1:** Half-maximal inhibitory concentration (IC50 values) from neuroblastoma cell lines treated with quarfloxin and CX-5461

Cell line	*MYCN* status	*TP53* status	IC50 (nM)±SD	
			Quarfloxin	CX-5461
IMR-32	Amp.	Wt	5.0 ± 2.2	7.6 ± 0.21
CHP-134	Amp.	Wt	23.0 ± 14.7	40.1 ± 13.9
CHLA-15	Non-amp.	Wt	54.0 ± 3.7	25.8 ± 2.2
BE(2)-C	Amp.	Mut	84.3 ± 5.0	106.9 ± 2.4
Kelly	Amp.	Mut	150.1 ± 33.7	275.6 ± 29.7
SK-N-AS	Non-amp.	Mut	965.2 ± 122.8	747.5 ± 65.8
SK-N-FI	Non-amp.	Mut	967.6 ± 178.8	618.3 ± 99.1
SHEP-TET21N -dox	Exogenous, high MycN	Wt	20.2 ± 0.03	14.4 ± 2.8
SHEP-TET21N + dox	Exogenous, low MycN	Wt	357.2 ± 151.5	311.8 ± 9.1

Data from two independent experiments each consisting of two biological replicates per concentration. SD is the standard deviation between the individual experiments

These data show that quarfloxin and CX-5461 effectively inhibit the growth of neuroblastoma cells in vitro. MNA (or high *c-Myc*) and wt-*TP53* cell lines were found to be more sensitive to these drugs compared with cells with single-copy *MYCN* and inactivating *TP53* mutations.

### High MycN expression sensitizes neuroblastoma cell lines to quarfloxin and CX-5461

To further investigate the relationship between MycN expression and ribosome biogenesis in neuroblastoma cell lines, we reanalyzed microarray gene expression data from a previous *MYCN* siRNA experiment on IMR-32 cells performed by Bell et al. [15]. K-means clustering with all genes in the KEGG pathway “Ribosome Biogenesis in Eukaryotes” revealed two distinct clusters defined by a High-RiBi and a Low-RiBi group (Supplementary Figure 3A). The High- and Low-RiBi clusters consisted of samples with high *MYCN* expression (no siRNA, siSCR-16h, and siSCR-48 h) and low *MYCN* expression (siMYCN-16 h and siMYCN-48 h), respectively. The expression of *MYCN* was significantly higher in the High-RiBi cluster compared with the Low-RiBi cluster (Supplementary Figure 3B). These data show that siRNA-mediated knockdown of MycN expression represses the expression of genes involved in ribosome biogenesis.

To functionally investigate the role of MycN on the sensitivity of neuroblastoma cell lines to the ribosome biogenesis inhibitors, we utilized the SHEP-TET21N model system. SHEP-TET21N is a MycN-inducible cell line derived from the parental single-copy *MYCN* human neuroblastoma cell line SHEP [16]. In the presence of 1 μg/mL doxycycline (dox), SHEP-TET21N cells express undetectable levels of MycN, whereas removal of dox induces high MycN expression (Fig. 2b, insert).

High MycN SHEP-TET21N cells grown without dox had a lower IC_50_ value for quarfloxin and CX-5461 compared with their low MycN expressing counterparts treated with 1 µg/mL doxycycline (Fig. 2b, Table 1).

Next, we used two different siRNAs targeting *MYCN* to knockdown MycN expression in the MNA neuroblastoma cell line IMR-32. Cells were transfected with siMYCN-1 and siMYCN-2 (siRNAs targeting *MYCN*) or siNC (a negative control siRNA), and treated with CX-5461 and quarfloxin. Efficient MycN knockdown was confirmed by western blot (Supplementary Figure 4A). After 48 h of drug treatment, both siMYCNs repressed the cytotoxic effect of the drugs when compared with siNC-treated cells (Fig. 2c). Similar results were observed in CHP-134 cells (Supplementary Figure 5).

These data confirm that neuroblastoma cells with high MycN expression are more sensitive to CX-5461 and quarfloxin than cells with low MycN expression.

### Quarfloxin and CX-5461 induce DNA damage, p53 signaling, cell death, and cell cycle arrest in neuroblastoma cell lines

**Fig. 3:**
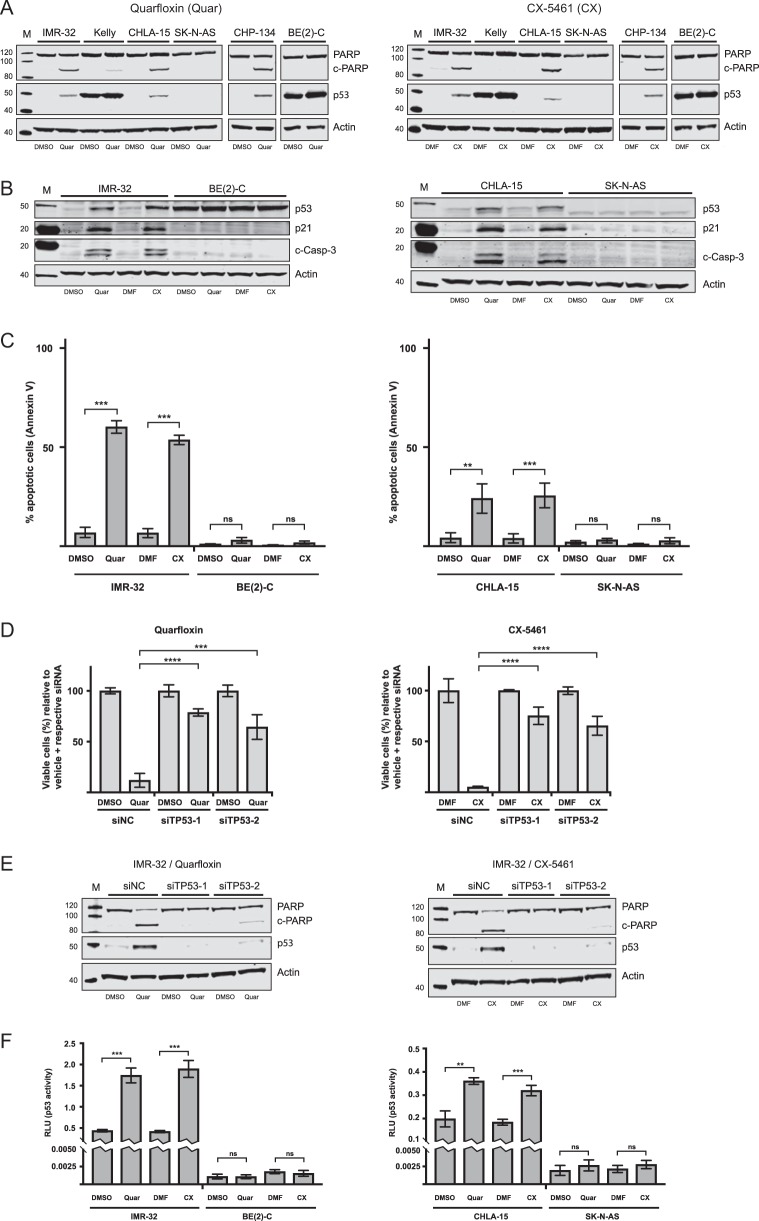
Quarfloxin and CX-5461 induce p53-dependent apoptosis in *TP53*-wt neuroblastoma cells. **a** Representative western blot (WB) for the assessment of cleaved PARP (c-PARP) and p53 expression in neuroblastoma cell lines treated for 24 h with 150 nM quarfloxin (left panel) or 230 nM CX-5461 (right panel) or 0.1% vehicle (DMSO and DMF, respectively). **b** Representative WB evaluation of cleaved-Caspase-3 (c-Casp-3), p53, and p21 expression in neuroblastoma cell lines treated as in **a**. **c** FACS analysis of Annexin V-positive neuroblastoma cells after 24 h treatment with 150 nM quarfloxin or 230 nM CX-5461 or vehicle. The data represents the mean and SD of representative experiments performed with two biological replicates pr. condition (ns, not significant; **p ≤ 0.01; ***p ≤ 0.001). **d** p53 induction sensitizes neuroblastoma cells to quarfloxin and CX-5461. The day after transfection with siRNAs targeting *TP53* (siTP53-1 and siTP53-2), 50 nM quarfloxin (left panel) or 75 nM CX-5461 (right panel) were added and cell viability was measured 48 h after treatment. The viability of vehicle + respective siRNA was set to 100%, and quarfloxin and CX-5461 treated cells were normalized against their respective controls. The data represents the mean cell viability and s.d. of two individual experiments performed in duplicate (***p ≤ 0.001; ****p ≤ 0.0001). **e** Representative WB assessing c-PARP levels after p53 knockdown. IMR-32 cells were reverse transfected with siTP53-1 and siTP53-2, and treated with 150 nM quarfloxin (left panel) or 230 nM CX-5461 (right panel) for 24 h before harvesting and immunoblotting. **f** Assessment of p53 transcriptional activity. Neuroblastoma cells were co-transfected immediately after seeding with a p53-responsive firefly luciferase reporter (pg13) and CMV-renilla luciferase. 150 nM quarfloxin or 230 nM CX-5461 were added the following day and cells were treated for 24 h before harvesting and analysis. Pg-13 expression was normalized to Renilla expression (RLU) and the data shown represents the mean of one experiment with three biological replicates and is representative of two independent experiments (ns, not significant; **p ≤ 0.01; ***p ≤ 0.001)

To investigate the mechanisms underlying the observed growth repression of neuroblastoma cells treated with quarfloxin and CX-5461, we analyzed several cell lines for the presence of apoptotic markers. First, we assessed the presence of the 89 kDa cleaved PARP (c-PARP) fragment and p53 on western blots of protein extracts isolated from cells treated with 150 nM quarfloxin or 230 nM of CX-5461 or 0.1% vehicle (DMSO and DMF, respectively). For all the wt-*TP53* cell lines tested (IMR-32, CHP-134, and CHLA-15), we detected increased presence of c-PARP and induction of the p53 protein after 24 h of treatment (Fig. 3a). On the contrary, mut-*TP53* cells (Kelly, SK-N-AS, and BE(2)-C) showed no significant increase in c-PARP or p53 expression. We further confirmed these observations by showing that two representative wt-*TP53* neuroblastoma cells (IMR-32 and CHLA-15) treated with quarfloxin or CX-5461 had increased expression of the 17/19 kDa cleaved-Caspase-3 (c-Casp-3) and induction of p21 protein and mRNA expression (Fig. 3b, Supplementary Figure 6). Enhanced c-Casp-3 or p21 expression was not observed in the mut-*TP53* neuroblastoma cell lines BE(2)-C and SK-N-AS treated with quarfloxin or CX-5461 (Fig. 3b).

Next, we used flow cytometry to analyze representative wt-*TP53* and mut-*TP53* neuroblastoma cell lines for the apoptotic marker Annexin V after treatment with quarfloxin or CX-5461. As shown in Fig. 3c, an increase in the proportion of Annexin V-positive cells after treatment with quarfloxin or CX-5461 was only observed in the wt-*TP53* cell lines IMR-32 and CHLA-15.

To further confirm the importance of functional p53 on the cytotoxic effect observed upon exposure of neuroblastoma cell lines to quarfloxin and CX-5461, we performed knockdown of *TP53* using two siRNAs (siTP53-1 and siTP53-2, Supplementary Figure 4B). When p53 expression was repressed by siTP53-1 and siTP53-2 in IMR-32 cells, the cytotoxic effect of quarfloxin and CX-5461 was reduced (Fig. 3d) and c-PARP was almost completely abolished (Fig. 3e). A similar, but less prominent, reduction in cell viability upon p53 knockdown was observed in CHP-134 cells (Supplementary Figure 5). Induction of functional p53 signaling by quarfloxin or CX-5461 was validated using a p53-responsive luciferase assay [17]. Only the wt-*TP53* cell lines (IMR-32 and CHLA-15) showed a significant increase in luciferase activity, indicating induction of functional p53 expression in cells exposed to CX-5461 or quarfloxin (Fig. 3f).

However, when we reconstituted functional p53 in BE(2)-C cells by exogenous overexpression of wt-p53, we did not observe a rescue in viability (Supplementary Figure 7A) or increased apoptosis (Supplementary Figure 7B) upon treatment with quarfloxin or CX-5461.

**Fig. 4:**
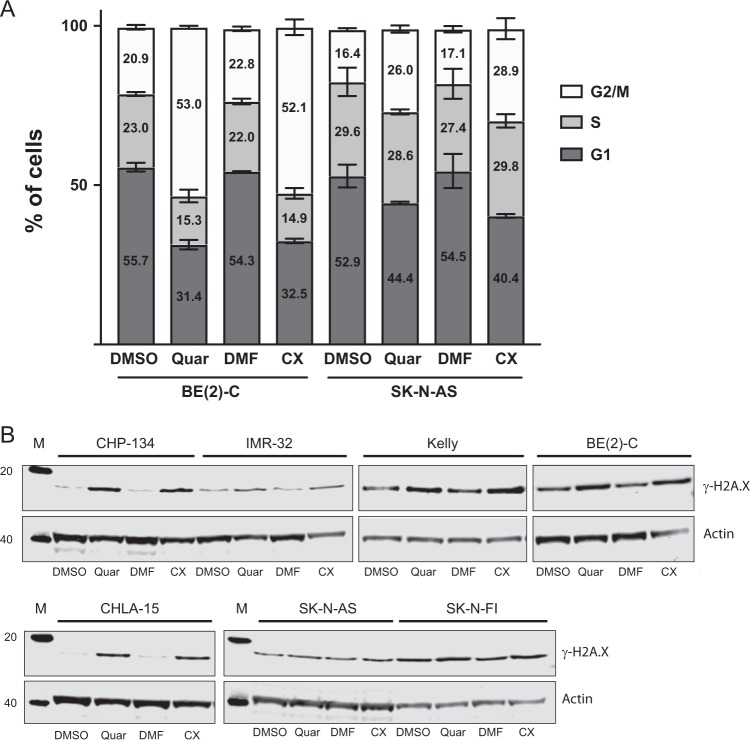
Analysis of cell cycle distribution and DNA damage in neuroblastoma cell lines. **a** Quarfloxin and CX-5461 induce G2/M-arrest in *TP53*-mutated neuroblastoma cell lines. Flow cytometry analysis of cell cycle distribution in neuroblastoma cell lines BE(2)-C and SK-N-AS after 24 h treatment with 150 nM quarfloxin or 230 nM CX-5461. The data represent the mean percentage and SD of cells in G1, S, and G2/M phases from two individual experiments, each performed with two biological replicates. **b** Quarfloxin and CX-5461 induce DNA damage in neuroblastoma cell lines. Representative western blot of DNA damage marker γ-H2A.X in neuroblastoma cell lines exposed to 150 nM quarfloxin or 230 nM CX-5461 for 24 h

To further understand the growth impairing effects of quarfloxin and CX-5461 in the cell lines failing to undergo apoptosis, we evaluated cell cycle distribution profiles in BE(2)-C and SK-N-AS cells after treatment with these compounds. Both cell lines showed an accumulation in the G2/M-phase of the cell cycle after 24 h exposure to the drugs (Fig. 4a).

Genotoxic stress is a major activator of p53 signaling, therefore we investigated whether quarfloxin and CX-5461 have the propensity to induce DNA damage in neuroblastoma cell lines. In order to assess this, we evaluated the presence of DNA damage biomarker γ-H2A.X on western blots from several neuroblastoma cell lines. In all cell lines tested, we detected an increase of this marker, compared with vehicle-treated cells (Fig. 4b).

Together these results show that CX-5461 and quarfloxin induced DNA damage and impaired growth of neuroblastoma cells by apoptosis in wt-*TP53* cells and G2/M-arrest in mut-*TP53* cells.

### Quarfloxin and CX-5461 reduce the expression of MycN and pre-rRNA (47S-rRNA)

**Fig. 5:**
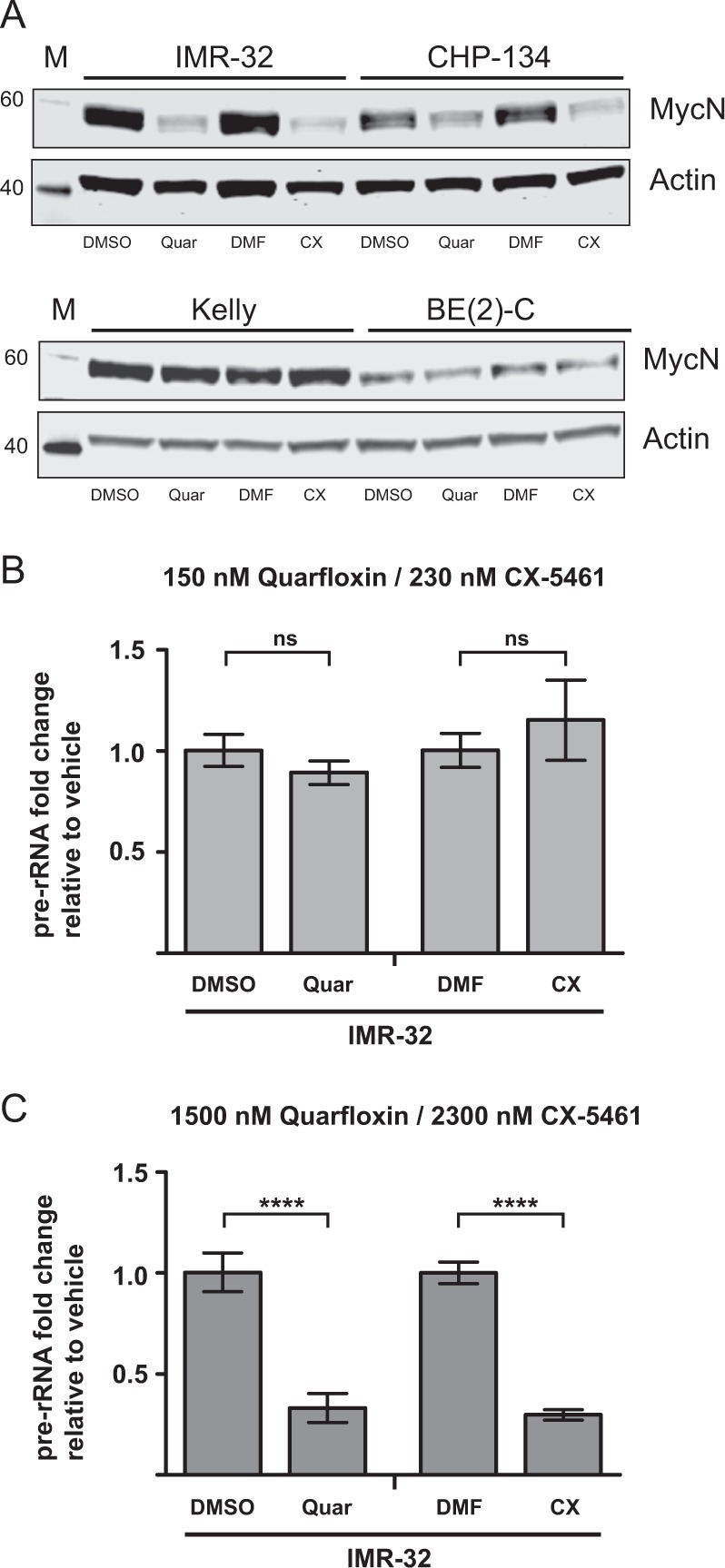
Analysis of MycN and pre-rRNA expression in neuroblastoma cells treated with quarfloxin and CX-5461. **a** The protein levels of MycN are depleted in neuroblastoma cells with functional p53 upon treatment with quarfloxin or CX-5461. Representative western blot showing MycN protein levels in wt-*TP53* IMR-32 and CHP-134 (upper panel) and *TP53*-mutated Kelly and BE(2)-C (lower panel) cells after 48 h treatment with 150 nM quarfloxin or 230 nM CX-5461. **b** Expression of 47S-rRNA (pre-rRNA) in IMR-32 cells treated for 24 h with low doses of quarfloxin (150 nM) and CX-5461(230 nM). **c** Expression of 47S-rRNA (pre-rRNA) in IMR-32 cells treated for 24 h with high doses of quarfloxin (1500 nM) and CX-5461(2300 nM). ns, not significant; ****p ≤ 0.0001

To further explore how the RNA pol I inhibitors influence the biology of *MYCN*-amplified neuroblastoma cells, we examined the effect of quarfloxin and CX-5461 on MycN expression in several different *MYCN*-amplified neuroblastoma cell lines. MycN was downregulated in IMR-32 and CHP-134 cells, but not in mut-*TP53* BE(2)-C and Kelly cells after 48 h of drug treatment (Fig. 5a).

As quarfloxin and CX-5461 originally were characterized as direct inhibitors of RNA pol I activity, we assessed the ability of quarfloxin and CX-5461 to suppress the expression of RNA pol I transcript 47S-rRNA. The 47S-rRNA contains the 5’ETS of rDNA genes, which is the first region to be processed with fast kinetics, and is therefore often used as a proxy for the pre-rRNA transcriptional activity of RNA pol I [18]. To our surprise, quarfloxin and CX-5461 concentrations, which effectively induced DNA damage, cell death, p53 signaling, and cell cycle arrest, did not downregulate the expression of 47S-rRNA after 24 h of treatment (Fig. 5b). However, a downregulation of 47S-rRNA was observed after 24 h with a 10-fold increase of the quarfloxin or CX-5461 doses (Fig. 5c). In order to investigate other components of the ribosomal subunits, we measured the expression of mature rRNAs (18S-, 5.8S-, and 28S-rRNA) and a subset of ribosomal proteins (RPL13A, RPL32, RPS5, and RPS19) in IMR-32 cells exposed to low and high doses of quarfloxin or CX-5461. Similar to our observations of 47S-rRNA expression, no significant changes were observed for the other components of ribosomal subunits when IMR-32 cells were exposed to the low cytotoxic doses of the inhibitors, except a minor reduction in expression of RPL32. However, when the same cells were exposed to 10-fold higher doses of quarfloxin and CX-5461, we measured increased levels of 18S-rRNA and 28S-rRNA. A similar increase was observed for ribosomal proteins RPL13A and RPS5 (Supplementary Figure 8).

### CX-5461 represses the growth of neuroblastoma xenografts

**Fig. 6:**
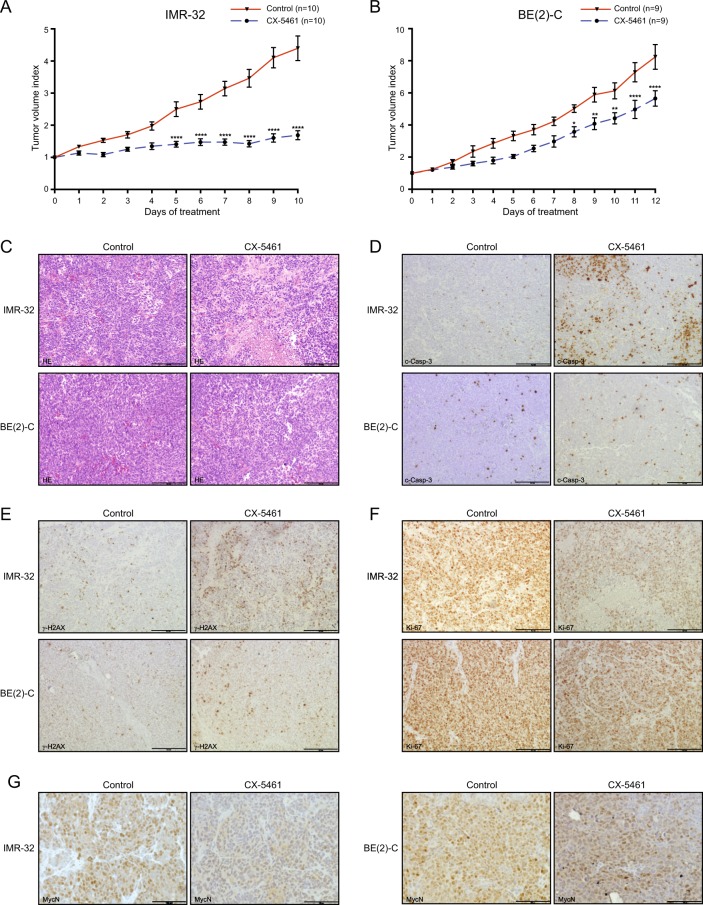
CX-5461 inhibits neuroblastoma xenograft growth in vivo. **a** Comparison of tumor growth in IMR-32 xenograft bearing mice receiving no treatment (*n* = 10) or CX-5461 (*n* = 10) for 3–6 consecutive days and then every third days for 10 days. Tumor volume index (TVI) means with SEM is displayed (****p ≤ 0.0001). **b** Comparison of tumor growth (tumor volume index; TVI) in BE(2)-C engrafted mice receiving no treatment (*n* = 9) or CX-5461 (*n* = 9) every third day for 12 days. TVI means with SEM is displayed (*p ≤ 0.05; **p ≤ 0.01; ****p ≤ 0.0001). **c** Sections of dissected tumors were analyzed by hematoxylin and eosin (H&E) staining. IMR-32 xenografts sections deriving from mice treated with CX-5461 demonstrated areas with necrotic tissues, which was less evident in corresponding BE(2)-C xenografts, and **d** immunohistochemical staining with antibodies to detect the apoptotic marker c-Casp-3, **e** the DNA damage marker γ-H2A.X, **f** the proliferation marker Ki-67, and **g** MycN. Scalebar = 100 μm

Since quarfloxin and CX-5461 show almost identical effects in all neuroblastoma cell lines tested and because CX-5461 is currently in clinical trials, we decided to limit our preclinical in vivo investigations to CX-5461. In order to evaluate the therapeutic potential of CX-5461 in vivo, NMRI *nu/nu* mice with established IMR-32 or BE(2)-C xenograft tumors were treated by oral administration of CX-5461. In both models, a significant decrease of tumor growth was observed, with treated IMR-32 tumors reduced to 38% (Fig. 6a) and BE(2)-C to 69% (Fig. 6b) at the end of treatment compared with their respective control groups.

Histological analysis of tissue sections from treated tumors revealed a pale appearance with regional necrosis, hemorrhage and significant increase in damaged cells with disintegrated cytoplasm (Fig. 6c, Supplementary Figure 9). In addition, higher number of apoptotic cell bodies and fragmented nuclei were evident as confirmed by immunohistochemical detection of c-Casp-3 (Fig. 6d) and γ-H2A.X (Fig. 6e) levels in tumors from mice treated with CX-5461 compared with control tumors. Furthermore, compared with control tumors, treatment was associated with a prominent reduction in staining of the cell proliferation marker Ki-67 (Fig. 6f) and reduced levels of MycN protein (Fig. 6g). These histological features were in particular evident in tumor tissue from the *MYCN*-amplified IMR-32 xenograft. The data suggest an inhibitory effect of CX-5461 on tumor proliferation by induction of a DNA damage response and apoptosis.

## Discussion

Despite intensive treatment modalities, the survival of patients with high-risk neuroblastoma is still around 50%. The major marker attributed to high-risk neuroblastoma is *MYCN* gene amplification and high-risk neuroblastoma patients without *MYCN*-amplification frequently exhibit high expression of either MycN, c-Myc, or Myc signature genes [19]. Our analysis of publicly available gene expression RNAseq and microarray data, show that high-risk neuroblastoma with high *MYCN* expression strongly correlates to elevated expression of genes involved in ribosome biogenesis and poor patient survival. Several observations have shown that c-Myc is an important regulator of ribosome biogenesis by affecting the expression of ribosomal genes as well as auxiliary factors important in ribosome biogenesis [7]. Also, elevated MycN expression has been suggested to augment the expression of several genes involved in ribosome biogenesis and protein synthesis [6]. Similarly, we found that siRNA-mediated downregulation of MycN expression resulted in reduced expression of genes involved in ribosome biogenesis. Hyperactive ribosome biogenesis, which morphologically can be detected by an increase in nucleolar size and number, is a hallmark for the majority of cancers and is frequently associated with poor prognosis [5]. In neuroblastoma, poorly differentiated cells exhibit increased number of nucleolar organizer regions [20] suggesting increased proliferation and elevated ribosome biogenesis. Furthermore, neuroblastomas with high expression of MycN or c-Myc have been shown to exhibit enlarged nucleoli [8, 9]. Together, these data suggest that inhibition of ribosome biogenesis in neuroblastoma should be tested as a treatment option for neuroblastoma patients expressing high levels of MycN and/or c-Myc. In the present study, we show that treatment with quarfloxin or CX-5461, two small molecule inhibitors of RNA polymerase I, inhibited the growth of neuroblastoma cells with IC_50_ at nanomolar concentrations. Neuroblastoma cells expressing high levels of *MYCN* or *c-MYC* were significantly more sensitive to treatment with these compounds. We also show that both drugs reduce the levels of MycN protein and that wt-*TP53* neuroblastoma cell lines activate p53 signaling and undergo apoptosis, whereas mut-*TP53* cells undergo cell cycle arrest in the G2/M-phase. Previously, CX-5461 has been shown to prevent initiation of rRNA synthesis by RNA polymerase I through inhibiting the binding of the transcription factor SL1 to the rDNA promoter, with subsequent release of free ribosomal proteins and activation of the nucleolar stress pathway promoting the activation of p53 signaling [21]. Exogenous addition of wt-*TP53* cDNA by transient transfection did not result in increased effects of CX-5461 or quarfloxin in BE(2)-C cells, a neuroblastoma cell line expressing high levels of mutant *TP53* (*TP53mut C135F*). *TP53*-C135F lies within the DNA-binding domain of the p53 protein (UniProt.org) and confers a loss of function to the p53 protein as demonstrated by loss of p53 transactivation activity [22]. In fact, the C135F-mutated version of p53 from SK-N-BE(2) cells has been shown to exert a moderate dominant negative effect on wt-p53 transcriptional activity [23]. Hence, the reason for the lack of increased effects of CX-5461 or quarfloxin in BE(2)-C could be caused by the presence of high levels of endogenous mutant-p53 protein that interferes with the exogenous wt-p53 during complex formation and binding to DNA, resulting in an incomplete restoration of the transactivation activity [24]. This also partly explains that we observed an increase in p21 expression in wt-*TP53* transfected cells but no increase in apoptotic marker (c-PARP) or rescue of cell viability. Together, this can, at least partly, explain the differential effects observed in neuroblastoma cells containing wt-*TP53* versus those cells with mutated *TP53*.

Destabilization of MycN or c-Myc proteins mediated by RNA polymerase I inhibitors has also been established by others [25, 26]. In our study, we did not observe reduction of MycN expression in the *TP53*-mutated cell lines Kelly and BE(2)-C, whereas MycN was robustly downregulated in wt-*TP53* IMR-32 and CHP-134 (Fig. 5a). A similar observation was very recently reported by Niemas-Teshiba et al. [21]. In this study, a low dose of CX-5461 (250 nM) efficiently repressed MycN expression in wt-*TP53* LAN5 cells after 24 h exposure, whereas a fourfold higher dose (1000 nM), only led to a very slight suppression of MycN in *TP53*-mutated Kelly cells. This differential response to the drugs with regards to MycN expression could be explained by several possible mechanisms. Kelly and BE(2)-C cells have been characterized as drug-resistant cell lines whereas IMR-32 and CHP-134 are drug sensitive [27–29]. Therefore, the difference in MycN downregulation caused by CX-5461 and quarfloxin might be due to enhanced drug efflux caused by high levels of transporter molecules present in Kelly and BE(2)-C cells. In fact, the multifunctional drug transporter protein RALBP1/RLIP76 has been shown to be repressed by p53 in neuroblastoma cells expressing wt-p53. In contrast, SK-N-BE(2) cells expressing mutant-p53 showed overexpression of RALBP1/RLIP76 and increased drug resistance [30]. Another mechanism contributing to the difference in MycN expression could be explained by effects on the MycN degradation machinery. The FBXW7 E3-ubiquitin ligase is an important regulator of MycN stability in neuroblastoma cells [31]. In a study by Burmakin et al., reactivation of p53 by RITA was shown to induce a rapid and substantial downregulation of MycN via FBXW7-mediated proteasomal degradation in neuroblastoma cells [31]. Furthermore, reports from both ovarian and gastric cancers have shown that FBXW7 is downregulated in *TP53*-mutated tumors [32, 33]. When a dominant negative (R175H) p53 was overexpressed in wt-*TP53* ovarian cancer cell lines, the expression of FBXW7 was suppressed.

Interestingly, the *c-MYC* gene contains a G-quadruplex motif in its gene promoter and *MYCN* contains a G-quadruplex in intron 1, a region shown to be important for the transcriptional activation of this gene [34–36]. Quarfloxin has previously been described as a G-quadruplex stabilizer and reduces rRNA synthesis through disruption of the interaction between putative G-quadruplex structures in the rDNA and nucleolin [13]. Stabilization of G-quadruplexes in promoter regions has been shown to inhibit transcription, thereby providing another potential mechanism of *MYC* gene suppression by quarfloxin and CX-5461 [37].

Both quarfloxin and CX-5461 have previously been described as RNA polymerase I inhibitors [12, 13]. In our experiments, we did not observe any change in expression of 47S-rRNA when IMR-32 cells were exposed to quarfloxin and CX-5461 at doses which effectively induced DNA damage, cell death, p53 signaling, and cell cycle arrest (Fig. 5b). In addition, the expression of other components of the ribosomal subunits (mature rRNAs and ribosomal proteins) was also unaffected by this treatment (Supplementary Figure 8A and 8C). We therefore conclude that treatment with low cytotoxic doses of quarfloxin and CX-5461 does not significantly inhibit ribosomal biogenesis in *MYCN*-amplified neuroblastoma cell lines. However, when the cells were exposed to 10-fold higher doses of the drugs, we observed a marked downregulation of 47S-rRNA expression (Fig. 5c). These high concentrations of quarfloxin and CX-5461 also increased the levels of 18S-rRNA, 28S-rRNA, RPL13A, and RPS5 (Supplementary Figure 8B and 8D). We do not have a reasonable biological explanation for the discrepancy observed during exposure of high doses of the drugs. From the raw Cq values, we observe a significant increase, indicating reduced levels, of the mRNA housekeeping genes (*SDHA* and *ACTB*), 5.8S- and 47 S rRNA when cells were exposed to high doses of the drugs. This may explain the apparent increase of 18S- and 28S-rRNA measurements as a normalization artifact, rather than increased expression of these highly expressed and very stable rRNAs. A similar observation was reported in a study by Xu et al., demonstrating that these drugs caused synthetic lethality in BRCA1/2-deficient breast and ovarian cancers at doses which did not inhibit RNA pol I activity [38]. This study uncovered a novel mechanism of action for these drugs through causing replication-dependent ssDNA damage and subsequent growth arrest and apoptosis by stabilization of G-quadruplex structures in the genomic DNA. We demonstrate DNA damage in all neuroblastoma cell lines tested after treatment with quarfloxin and CX-5461, and in vivo DNA damage after CX-5461 treatment of nude mice bearing neuroblastoma xenografts. Our findings suggest that quarfloxin and CX-5461 cause cellular toxicity through a process involving stabilization of G-quadruplexes. The initial characterization studies by Drygin et al. described quarfloxin and CX-5461 as non-genotoxic using the Ames and Comet assay [12, 13]. This is in contrast to our results and the findings of Xu et al. [38] Furthermore, reports by Negi et al. and Quin et al. have shown that CX-5461 induces the ATM/ATR signaling pathway leading to a G2/M-phase cell cycle arrest [39, 40]. ATM and ATR are crucial sensors of DNA damage and activate the DNA damage response upon the presence of dsDNA or ssDNA breaks, respectively [41]. Negi et al. [39] demonstrated the enhancement of apoptosis in leukemia cells when co-treating with VE-822, an ATR inhibitor, and CX-5461. These results suggest that CX-5461 induces ssDNA damage, and show that co-targeting DNA damage response pathways can be utilized to overcome potential resistance to this agent.

CX-5461 significantly inhibited the growth of established *MYCN*-amplified neuroblastoma grown as xenografts in nude mice. Growth suppression was more pronounced in tumors harboring wt-*TP53* (IMR-32) compared with neuroblastoma containing mutated *TP53* (BE(2)-C). These results further support the notion that RNA polymerase I inhibitors activate p53 signaling and that p53 activation is one mechanism for the growth inhibiting effects seen by RNA polymerase I inhibitors in tumor cells.

Taken together, RNA polymerase I inhibitors are promising agents that should be further investigated in clinical studies as a treatment options for neuroblastoma patients with high expression of MycN or c-Myc.

## Materials and methods

### Cell culture

All cells were grown in a humidified incubator at 37 °C with 5% CO_2_. BE(2)-C, SK-N-AS, CHP-134, Kelly and SHEP-TET21N cells were maintained in RPMI-1640 supplemented with 10% sterile-filtered fetal bovine serum (FBS). IMR-32 and SK-N-FI were grown in low glucose DMEM supplemented with 10% FBS and 1% non-essential amino acids (NEAA). CHLA-15 cells were grown in IMDM with 20% FBS, 1x ITS, and 4 nM L-glutamine. All cell identities were confirmed by short tandem repeat analysis, and the cells were regularly checked to be mycoplasma-free.

### Chemicals

CX-5461 (Selleckchem) was resuspended in dimethylformamide (DMF) to a 5 mM stock. Quarfloxin (AdooQ) was resuspended in DMSO to a stock of 10 mM as recommended by the manufacturers. Aliquots of both chemicals were kept at −80 °C and were thawed and diluted in their respective vehicles (DMF or DMSO) for working solutions. The concentration of vehicle never exceeded 0.1%. For animal studies, CX-5461 was dissolved in 50 mM NaH_2_PO_4_ (pH 4.3) at 5 mg/mL.

### Viability assays

For IC_50_ experiments, cells grown in 24-well plates were treated with an 8-log dose range of quarfloxin and CX-5461 in a total volume of 500 μL/well for 48 h before the addition of 50 μL Alamar blue reagent (Thermo Fisher) directly to the wells. Cells were incubated at 37 °C for an additional 2 h and 100 μL of media was transferred to a black-walled 96-well plate and fluorescence was measured at 540 nm excitation and 590 nm emission wavelengths using a micro plate reader (CLARIOstar). The raw data was normalized to vehicle treated, and IC_50_ was calculated using the Prism 7 software and the equation: log(inhibitor) vs. normalized response—Variable slope. All experiments were repeated twice with two biological replicates.

### Flow cytometry

For cell cycle distribution profiling, cells were treated for 24 h in the presence of vehicle or 150 nM quarfloxin or 230 nM CX-5461. Floating and adherent cells (trypsination) were harvested and subsequently fixed and permeabilized in ice-cold 70% EtOH. Fixed cells were subsequently stained in PBS containing 50 μg/mL Propidium Iodide (PI) and 100 μg/mL RNaseA for 30 min protected from light at room temperature and then placed on ice. PI-stained cells were analyzed using a BD LSR Fortessa, and cell cycle data were further analyzed using FlowJo v.10 with the Watson model for evaluation of cell cycle distribution.

For the Annexin V apoptosis assay, cells were treated for 24 h in the presence of vehicle or 150 nM quarfloxin or 230 nM CX-5461 and analyzed using the FITC Annexin V Apoptosis Detection Kit according to the manufacturer’s instructions (BD Biopharmigen). Annexin V-positive cells were detected using a BD LSR Fortessa.

### Western blotting

Cells were treated with vehicle, quarfloxin, or CX-5461 as indicated. At harvesting, floating cells and adherent cells (trypsination) were collected for analysis. Protein isolation and blotting were performed essentially as previously described using NuPAGE 4–12% bis-tris precast polyacrylamide gels and Immobilon-PVDF membranes (Millipore) [42]. For western blots containing Caspase-3 and p21, 4–20% Tris-Glycine gels (Lonza) were used for better separation of these low MW proteins. Primary antibodies used in this study were mouse monoclonal anti-MycN (sc-53993, Santa Cruz Biotechnology, CA, USA), mouse monoclonal anti-p53 (sc-126, Santa Cruz Biotechnology, CA, USA), rabbit monoclonal anti-p21 (#2947, Cell Signaling Technology, MA, USA), rabbit polyclonal anti-PARP (#9542, Cell Signaling Technology, MA, USA), rabbit polyclonal anti-Caspase-3 (#9662, Cell Signaling Technology, MA, USA), mouse monoclonal anti-γ-H2A.X (05–636, Merck Millipore, MA, USA), rabbit polyclonal anti-actin (A2066, Sigma-Aldrich, MO, USA) and mouse monoclonal anti-actin (AB3280, Abcam, Cambridge, UK). Membranes were detected using the Odyssey Infrared Imaging system (LI-COR).

### RT-qPCR

Floating and adherent cells were lysed in 1 mL of Qiazol (Qiagen), 0.2 mL of chloroform was added for phase separation, and RNA was precipitated overnight from 0.4 mL of the aqueous phase 1:1 in isopropanol at −20 °C. The RNA pellet was washed twice in ice-cold 75% EtOH and resuspended in TE-buffer (10 mM Tris-HCl, 1 mM disodium EDTA, pH 8.0). RNA quality and quantity was assesed using the Nanodrop 2000. In total, 1200 ng of RNA was reverse transcribed using the High Capacity cDNA kit w/RNase inhibitor (Thermo Fisher). Each RT-qPCR reaction (20 μL) contained 12.5 ng of cDNA in 5 μL, 10 μL of Power SYBR (Thermo Fisher), 0.8 μL of 5 μM F+R primers, and 4.2 μL of nuclease-free H_2_O. Amplification of cDNA was carried out using a LightCycler 96 SW 1.1 (Roche). Relative expression of transcript levels was evaluated using the ddCT method with the geometric mean of two housekeeping genes (*SDHA* and *ACTB*). Expression of rRNA transcripts was normalized using the geometric mean of all rRNA transcripts according to [43]. Primer sequences were; SDHA: forward 5′-CTGATGAGACAAGATGTGGTG-3′, reverse 5′-CAATCTCCCTTCAATGTACTCC-3′, ACTB: forward 5′-CACCATGTACCCTGGCATT-3′, reverse 5′-ACGGAGTACTTGCGCTCAG-3′, p21: forward 5′-GCAGACCAGCATGACAGATTT-3′, reverse 5′-GGATTAGGGCTTCCTCTTGGA-3′, 47S-rRNA: forward 5′-CCGCGCTCTACCTTACCTAC-3′, reverse 5′-GCATGGCTTAATCTTTGAGACAAG-3′, 5.8S-rRNA: forward 5′-ACTCGGCTCGTGCGTC-3′, reverse: 5′-GCGACGCTCAGACAGG-3′, 18S-rRNA: forward 5′-GTAACCCGTTGAACCCCATT-3′, reverse 5′-CCATCCAATCGGTAGTAGCG-3′, 28S-rRNA: forward 5′-GGGTGGTAAACTCCATCTAAGG-3′, reverse 5′-GCCCTCTTGAACTCTCTCTTC-3′, RPL13A: forward 5′-TAAACAGGTACTGCTGGGCCG-3′, reverse 5′-CTCGGGAAGGGTTGGTGTTC-3′, RPL32: forward 5′-TACGACCCATCAGCCCTTGC-3′, reverse 5′-CATGATGCCGAGAAGGAGATGG-3′, RPS5: forward 5′-ATCATCAACAGTGGTCCCCG-3′, reverse 5′- AGATGGCCTGGTTCACACG-3′, RPS19: forward 5′-AAACCCCGTCGTTCCCTTTC-3′, reverse 5′ GCTTCCCGGACTTTTTGAGG-3′.

### p53 activity assays

Cells in 12-well plates were reverse transfected using Lipofectamine 2000 (Thermo Fisher) with 0.5 µg p53 transcriptional reporter (PG13) (Addgene), (originally published in Ref. [17]) and 0.02-μg pCMV-Renilla luc (Promega). On the next day, cells were treated with vehicles or 150 nM quarfloxin or 230 nM CX-5461. After 24 h of treatment, cells were collected in 200 μL 1x PLB buffer, and luciferase activity was measured using the Dual-Luciferase Reporter Assay according to the instructions from the manufacturer (Promega). PG13 firefly activity was normalized to renilla (RLU).

### siRNA transfections and *TP53* overexpression

Transfections were carried out using Lipofectamine 2000 and were performed essentially as described [44]. The following siRNAs were used (all from Qiagen): AllStars Negative Control siRNA (siNC), Hs_MYCN_4 FlexiTube siRNA (siMYCN_1), Hs_MYCN_6 FlexiTube siRNA (siMYCN_2), Hs_TP53_3 FlexiTube siRNA (siTP53_1), and Hs_TP53_9 FlexiTube siRNA (siTP53_2). Final concentrations of the respective siRNAs were 20 nM in all the experiments. The wt-p53 overexpression plasmid was a kind gift from Dr. Ugo Moens, University of Tromsø, Norway, empty vector was pCMV-XL4 (Origene). Final DNA plasmid concentration was 1 μg/mL of media.

### Xenografts

Four-to-six-week-old female Sca:NMRI *nu/nu* mice (Scanbur, Stockholm, Sweden) were maintained in pathogen-free conditions and given free access to sterile water and food. For BE(2)-C xenografts, 10 × 10^6^ cells were injected subcutaneously on the right flank under isoflurane anesthesia and for IMR-32 9 × 10^6^ cells in 50% Matrigel were injected following the same procedure. Tumors were measured every day with a digital caliper and tumor volume (mL) was calculated as volume = width^2^ × length × 0.44. When tumors reached ≥ 0.15 ml, mice were randomized into treatment with CX-5461 or control groups (no treatment). Mice in the treatment groups received 50 mg/kg CX-5461 by oral gavage every third day for 12 days for BE(2)-C or 3–6 consecutive days and then every third day for 10 days for IMR-32. Tumor volume index (TVI) was determined by dividing the tumor volume of each day by the starting volume (day 0). TVIs from treated and untreated tumors were compared in GraphPad Prism 7 using repeated measures two-way ANOVA with Bonferroni correction and *p*-values ≤ 0.05 were considered statistically significant. No significant weight changes were observed in both untreated and treated groups. At autopsy, tumors were fixed in 4% paraformaldehyde (PFA) at 4 °C for 24 h and then kept in 70% EtOH at 4 °C before further analysis. All animal experiments were approved by the regional ethics committee for animal research (N231/14), appointed and under the control of the Swedish Board of Agriculture and the Swedish Court. The animal experiments presented herein were in accordance with national regulations (SFS 1988:534, SFS 1988:539, and SFS 1988:541).

### Immunohistochemistry

Formalin-fixed and paraffin-embedded tissue sections were deparaffinized in xylene and graded alcohols, hydrated, and washed in a phosphate-buffered saline (PBS). After antigen retrieval in a sodium citrate buffer (pH 6) in a microwave oven, the endogenous peroxidase was blocked by 0.3% H_2_O_2_ for 15 min. Sections were incubated overnight at 4 °C with primary antibodies anti-γ-H2A.X (#9718 Cell Signal Technology, MA, USA), anti-cleaved Caspase-3 (Asp175) (Cell signaling Technology, MA, USA) or anti-Ki-67 (SP6, Neomarkers, CA, USA), respectively. As a secondary antibody, the anti-rabbit-horseradish peroxidase (HRP) SignalStain Boost IHC detection kit was used (# 8114, Cell Signaling Technology, MA, USA). For the assessment of MycN expression, a monoclonal mouse anti-MycN antibody was used (sc-53993, Santa Cruz Biotechnology, CA, USA). As a secondary antibody, anti-mouse EnVision-HRP (Dako, Agilent Technologies, Inc., Santa Clara, CA, USA) was used. A matched isotype control was also used as a control for nonspecific background staining.

### Statistical analysis

Differences between two groups were compared using the two-sided Student’s *t* test. Differences in treated and untreated xenograft tumor volume indexes were studied using repeated measures two-way ANOVA with Bonferroni correction. All statistical analysis was done using the GraphPad Prism 7 software and a result was considered statistically significant when *p* ≤ 0.05.

## supplementary information

Supplementary Table 1

Supplementary Figure 1

Supplementary Figure 2 A and B

Supplementary Figure 2 C and D

Supplementary Figure 3

Supplementary Figure 4

Supplementary Figure 5

Supplementary Figure 6

Supplementary Figure 7

Supplementary Figure 8

Supplementary Figure 9
