# Supplementary material for: Inhibitors of ribosome biogenesis repress the growth of MYCN-amplified neuroblastoma
Source: Oncogene. 2018 Dec 12;38(15):2800–13. doi: 10.1038/s41388-018-0611-7 (PMC6484764; doi:10.1038/s41388-018-0611-7)
Supplement: Supplementary file 1 — Supplementary Table 1 [file 41388_2018_611_MOESM1_ESM.docx]

**Supplementary Table 1:** Genetic characteristics of neuroblastoma cell lines used in this study

| **Cell line** | **Stage** | **Origin** | **Treatment** | ***MYCN*-amplified** | ***TP53*** | **1p del** | **11q del** | **17q gain** | **References** |
| --- | --- | --- | --- | --- | --- | --- | --- | --- | --- |
| BE(2)-C | 4 | Bone marrow | YES | YES | MUT | YES | NO | NO | [1, 2] |
| Kelly | 4 | Bone marrow | YES | YES | MUT | YES | YES | YES | [3, 4] |
| IMR-32 | unknown | Abdomen | NO | YES | WT | YES | YES | YES | [5] |
| CHP-134 | 4 | Lymph node  (primary: adrenal) | YES | YES | WT | YES | NO | YES | [6] |
| SK-N-FI |  | Bone marrow | YES | NO | MUT | NO | ? | ? | [8] |
| CHLA-15 | 4 | Primary | NO | NO | WT | ? | ? | ? | [7, 9] |
| SK-N-AS | 4 | Bone marrow  (primary: adrenal) | YES | NO | MUT | YES | YES | NO | [1, 10, 11] |
| *MYCN*-inducible neuroblastoma cell line: | | | | | | | | | |
| SHEP-Tet21N |  | Derived from SHEP (substrate adherent subtype of SK-N-SH) |  | +dox: OFF  -dox: ON | WT |  |  |  | [12] |

1. Thiele, C.J., *Neuroblastoma Cell Lines*, in *Neuroblastoma*, J.H.C. Culture. 1998: Lancaster, UK. p. 21-53.

2. Tweddle, D.A., A.J. Malcolm, N. Bown, A.D. Pearson*, et al.*, *Evidence for the development of p53 mutations after cytotoxic therapy in a neuroblastoma cell line.* Cancer Res, 2001. **61**(1): p. 8-13.

3. Gogolin, S., V. Ehemann, G. Becker, L.M. Brueckner*, et al.*, *CDK4 inhibition restores G(1)-S arrest in MYCN-amplified neuroblastoma cells in the context of doxorubicin-induced DNA damage.* Cell Cycle, 2013. **12**(7): p. 1091-104.

4. Piskareva, O., H. Harvey, J. Nolan, R. Conlon*, et al.*, *The development of cisplatin resistance in neuroblastoma is accompanied by epithelial to mesenchymal transition in vitro.* Cancer Lett, 2015. **364**(2): p. 142-55.

5. Tumilowicz JJ, Nichols WW, Cholon JJ, Greene AE. *Definition of a continuous human cell line derived from neuroblastoma*. Cancer Res. 1970 Aug;30(8):2110-8.

6. Schlesinger HR, Gerson JM, Moorhead PS, Maguire H, Hummeler K. *Establishment and characterization of human neuroblastoma cell lines*. Cancer Res. 1976 Sep;36(9 pt.1):3094-100.

7. Keshelava, N., R.C. Seeger, S. Groshen, and C.P. Reynolds, *Drug resistance patterns of human neuroblastoma cell lines derived from patients at different phases of therapy.* Cancer Res, 1998. **58**(23): p. 5396-405.

8. Helson, L., Nisselbaum, J., Helson, C., Majeranowski, A., and Johnson, G. A. *Biological markers in neuroblastoma and other pediatric neoplasias*. In: W. Davis, K. R. Harrap, and G. Stathopoulos (eds.), Human Cancer. Its Characterization and Treatment, pp. 86–94. Princeton: Excerpta Medica, 1980.

9. Keshelava, N., E. Davicioni, Z. Wan, L. Ji*, et al.*, *Histone deacetylase 1 gene expression and sensitization of multidrug-resistant neuroblastoma cell lines to cytotoxic agents by depsipeptide.* J Natl Cancer Inst, 2007. **99**(14): p. 1107-19.

10. Caren, H., H. Kryh, M. Nethander, R.M. Sjoberg*, et al.*, *High-risk neuroblastoma tumors with 11q-deletion display a poor prognostic, chromosome instability phenotype with later onset.* Proc Natl Acad Sci U S A, 2010. **107**(9): p. 4323-8.

11. Goldschneider, D., E. Horvilleur, L.F. Plassa, M. Guillaud-Bataille*, et al.*, *Expression of C-terminal deleted p53 isoforms in neuroblastoma.* Nucleic Acids Res, 2006. **34**(19): p. 5603-12.

12. Lutz W, Stohr M, Schurmann J, Wenzel A, Lohr A, Schwab M. *Conditional expression of N-myc in human neuroblastoma cells increases expression of alpha-prothymosin and ornithine decarboxylase and accelerates progression into S-phase early after mitogenic stimulation of quiescent cells.* Oncogene. 1996;13(4):803-12.
