# Supplementary figures and images for: Inhibitors of ribosome biogenesis repress the growth of MYCN-amplified neuroblastoma
Source: Oncogene. 2018 Dec 12;38(15):2800–13. doi: 10.1038/s41388-018-0611-7 (PMC6484764; doi:10.1038/s41388-018-0611-7)

SUPPLEMENTARY FIGURE 1

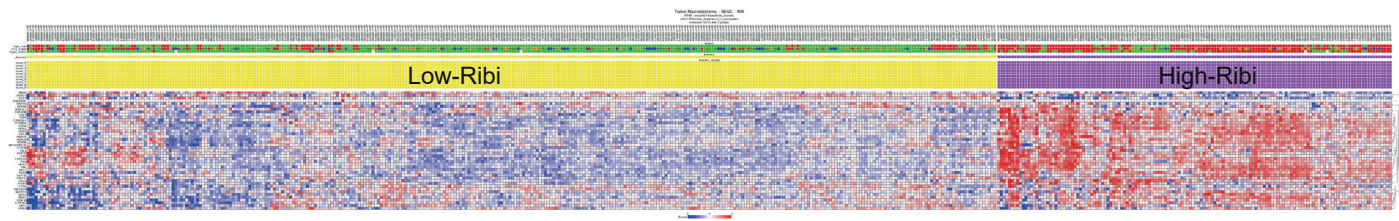

Supplement: Supplementary file 2 — Supplementary Figure 1 [file 41388_2018_611_MOESM2_ESM.pdf]

SUPPLEMENTARY FIGURE 3

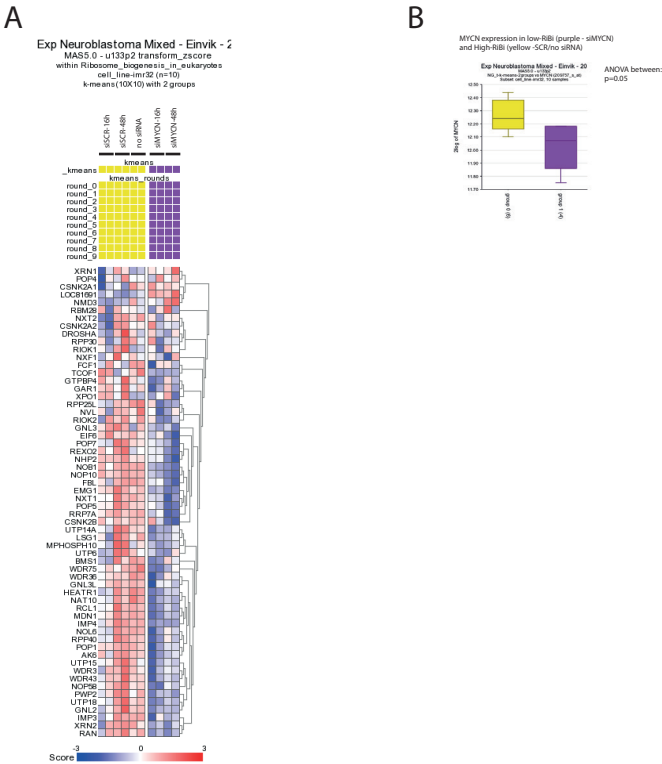

Supplement: Supplementary file 5 — Supplementary Figure 3 [file 41388_2018_611_MOESM5_ESM.pdf]

SUPPLEMENTARY FIGURE 4

A

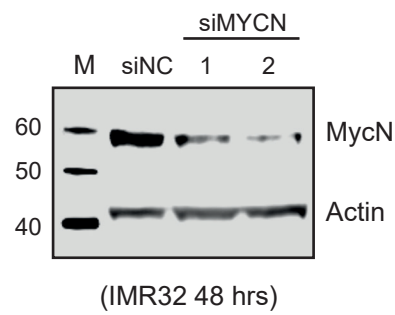

B

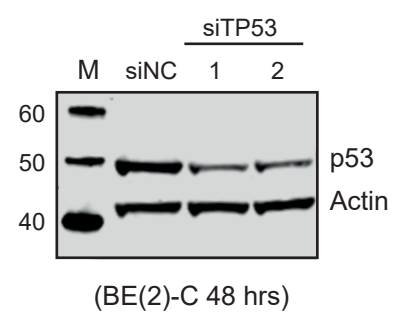

Supplement: Supplementary file 6 — Supplementary Figure 4 [file 41388_2018_611_MOESM6_ESM.pdf]

SUPPLEMENTARY FIGURE 5

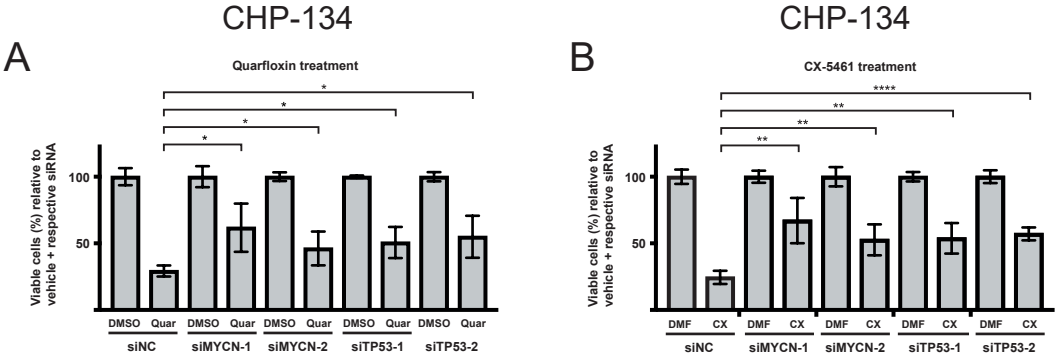

Supplement: Supplementary file 7 — Supplementary Figure 5 [file 41388_2018_611_MOESM7_ESM.pdf]

## SUPPLEMENTARY FIGURE 6

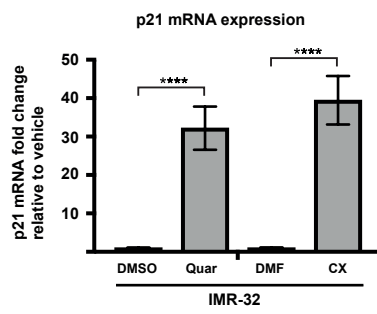

Supplement: Supplementary file 8 — Supplementary Figure 6 [file 41388_2018_611_MOESM8_ESM.pdf]

SUPPLEMENTARY FIGURE 7

BE(2)-C

A

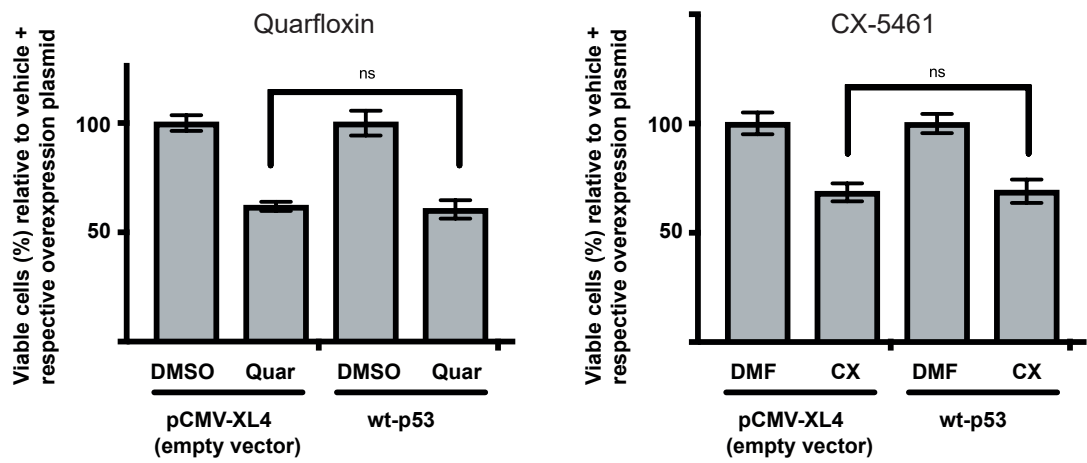

B

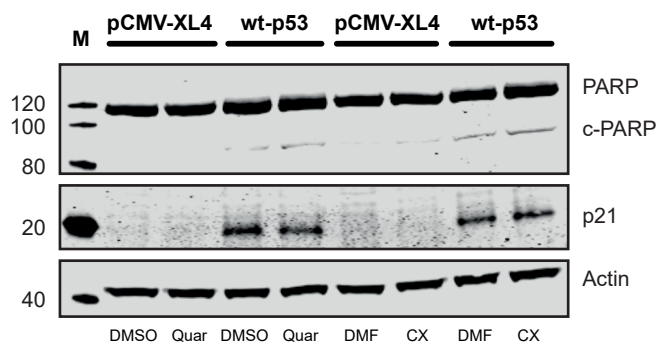

Supplement: Supplementary file 9 — Supplementary Figure 7 [file 41388_2018_611_MOESM9_ESM.pdf]

SUPPLEMENTARY FIGURE 8

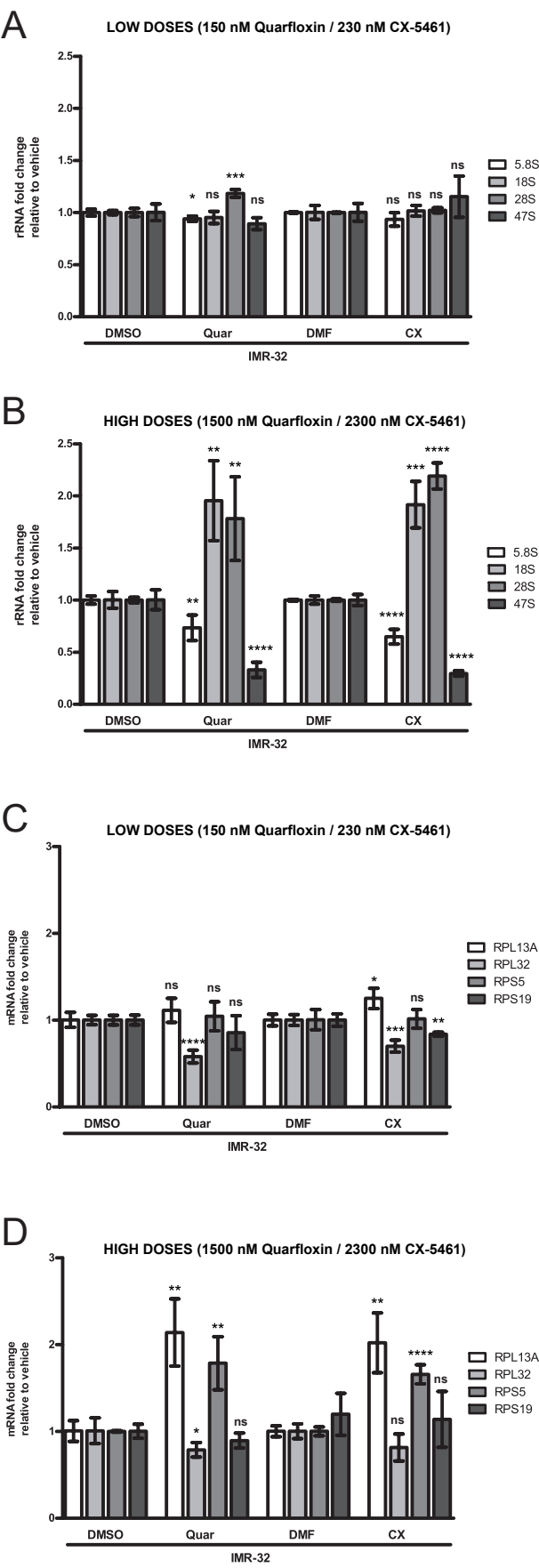

Supplement: Supplementary file 10 — Supplementary Figure 8 [file 41388_2018_611_MOESM10_ESM.pdf]

SUPPLEMENTARY FIGURE 9

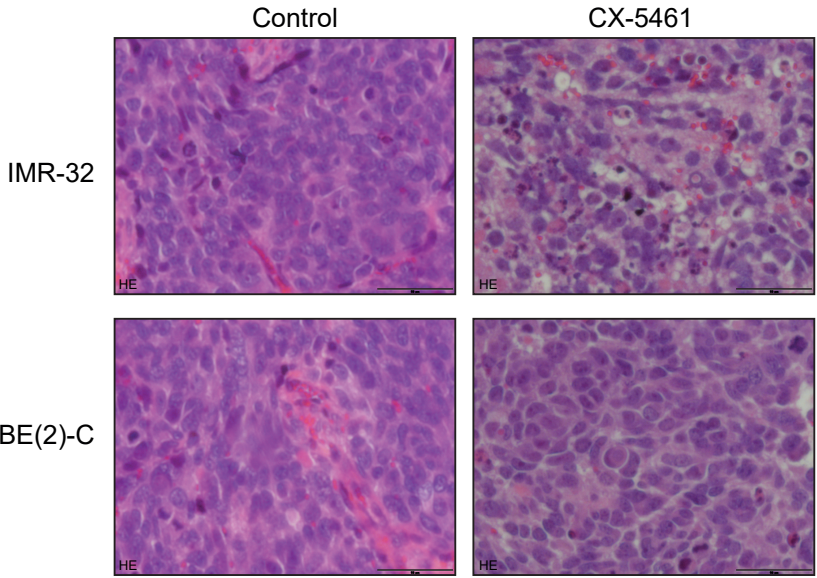

Supplement: Supplementary file 11 — Supplementary Figure 9 [file 41388_2018_611_MOESM11_ESM.pdf]
