# Supplementary material for: Inhibitors of ribosome biogenesis repress the growth of MYCN-amplified neuroblastoma
Source: Oncogene. 2018 Dec 12;38(15):2800–13. doi: 10.1038/s41388-018-0611-7 (PMC6484764; doi:10.1038/s41388-018-0611-7)
Supplement: Supplementary file 3 — Supplementary Figure 2 A and B [file 41388_2018_611_MOESM3_ESM.pdf]

SUPPLEMENTARY FIGURE 2

A

Neuroblastoma Versteeg-88

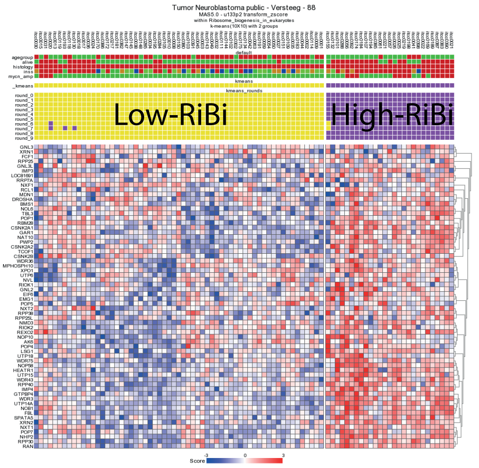

K-means clustering, 2 groups
“ribosome biogenesis in eukaryotes”

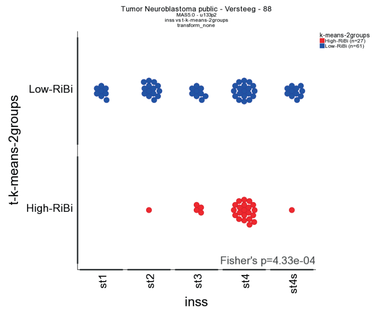

Distribution of High-RiBi and Low-RiBi tumors in INSS tumor stages

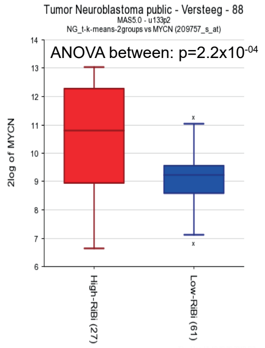

MYCN expression in High-RiBi and Low-RiBi tumors

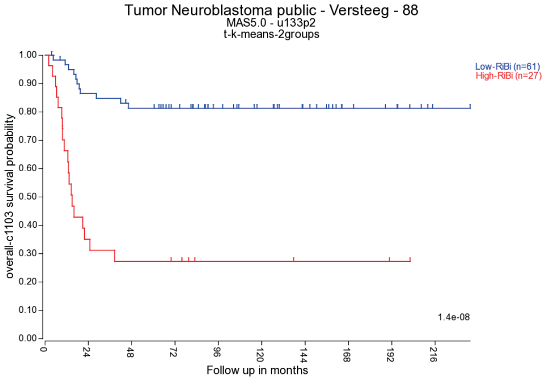

Overall survival between Low-Ribi and High-RiBi tumors

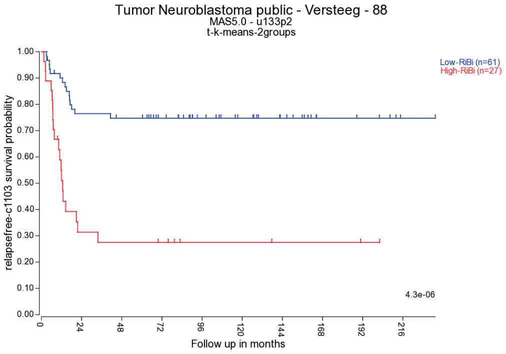

Relapsefree survival between Low-Ribi and High-RiBi tumors

B

Neuroblastoma Maris-101

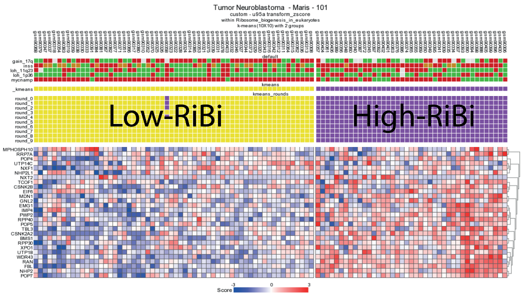

K-means clustering, 2 groups
“ribosome biogenesis in eukaryotes”

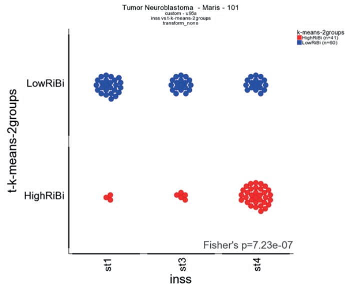

Distribution of High-RiBi and Low-RiBi tumors in INSS tumor stages

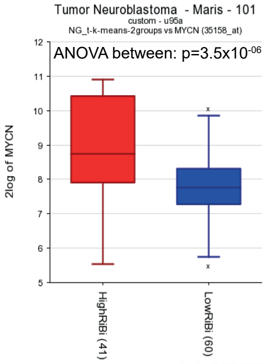

MYCN expression in High-RiBi and Low-RiBi tumors
