# Supplementary material for: Inhibitors of ribosome biogenesis repress the growth of MYCN-amplified neuroblastoma
Source: Oncogene. 2018 Dec 12;38(15):2800–13. doi: 10.1038/s41388-018-0611-7 (PMC6484764; doi:10.1038/s41388-018-0611-7)
Supplement: Supplementary file 4 — Supplementary Figure 2 C and D [file 41388_2018_611_MOESM4_ESM.pdf]

C

Neuroblastoma Oberthuer-251

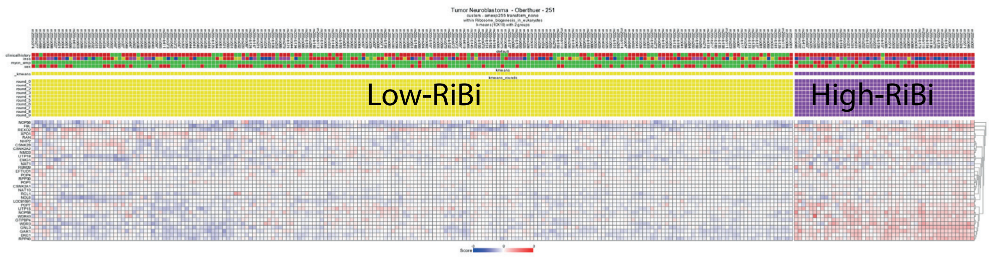

2 color array containing negative values.
To avoid filter out, K-mean's settings were as follow:
-floor value: -500
-range: 0
-max exp: -500

K-means clustering, 2 groups
"ribosome biogenesis in eukaryotes"

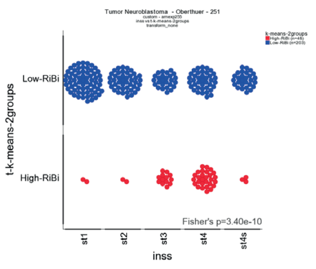

Distribution of High-RiBi and Low-RiBi tumors in INSS tumor stages

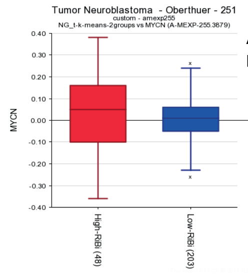

ANOVA between:
p=0.37

MYCN expression in High-RiBi and Low-RiBi tumors

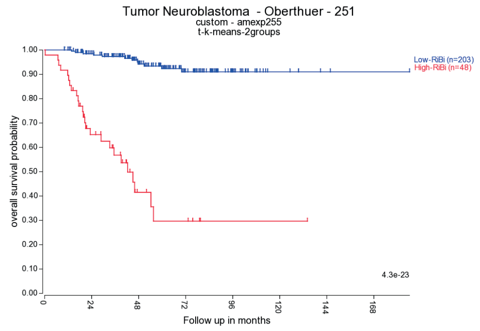

Overall survival between Low-Ribi and High-RiBi tumors

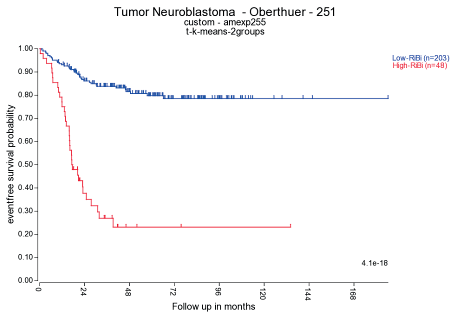

Eventfree survival between Low-Ribi and High-RiBi tumors

D

Neuroblastoma non-*MYCN*-amplified - Seeger-102

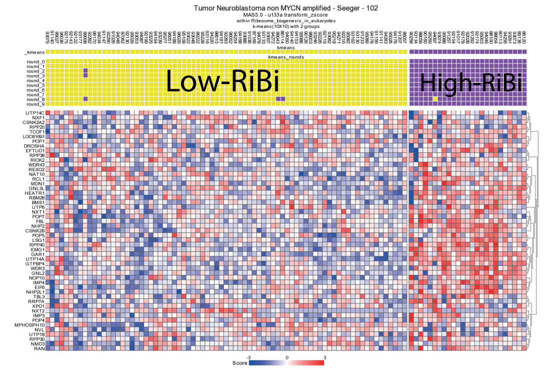

K-means clustering, 2 groups
"ribosome biogenesis in eukaryotes"

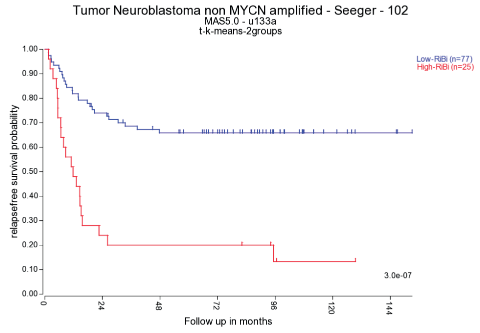

Relapsefree survival between Low-Ribi and High-RiBi tumors
